# Drug Repurposing: Tolfenamic Acid Inactivates PrbP, a Transcriptional Accessory Protein in *Liberibacter asiaticus*

**DOI:** 10.3389/fmicb.2016.01630

**Published:** 2016-10-18

**Authors:** Christopher L. Gardner, Fernando A. Pagliai, Lei Pan, Lora Bojilova, Maria I. Torino, Graciela L. Lorca, Claudio F. Gonzalez

**Affiliations:** Microbiology and Cell Science Department, Genetics Institute & Institute of Food and Agricultural Science, University of FloridaGainesville, FL, USA

**Keywords:** transcriptional accessory protein, *Liberibacter asiaticus*, tolfenamic acid, antimicrobial, citrus

## Abstract

CLIBASIA_01510, PrbP, is a predicted RNA polymerase binding protein in *Liberibacter asiaticus*. PrbP was found to regulate expression of a small subset of ribosomal genes through interactions with the β-subunit of the RNA polymerase and a short, specific sequence on the promoter region. Molecular screening assays were performed to identify small molecules that interact with PrbP *in vitro*. Chemical hits were analyzed for therapeutic efficacy against *L. asiaticus* via an infected leaf assay, where the transcriptional activity of *L. asiaticus* was found to decrease significantly after exposure to tolfenamic acid. Similarly, tolfenamic acid was found to inhibit *L. asiaticus* infection in highly symptomatic citrus seedlings. Our results indicate that PrbP is an important transcriptional regulator for survival of *L. asiaticus in planta*, and the chemicals identified by molecular screening assays could be used as a therapeutic treatment for huanglongbing disease.

## Introduction

*Liberibacter asiaticus* is the prevalent causative agent of huanglongbing (HLB; citrus greening), the most devastating citrus disease worldwide. Since its emergence in the early 1900's in China, *L. asiaticus* has been found in nearly every citrus producing region across the globe, with devastating consequences (Bove et al., 2006). In the United States, the economic impact of HLB has already been felt throughout the Florida citrus industry, where estimated losses have exceeded $9 billion since the pathogen was first detected in 2005; and the California citrus industry is preparing for similar devastation, as this pathogen continues to spread across the west coast (Gottwald, 2010).

*Liberibacter asiaticus* is a phloem-limited pathogen with uneven distribution throughout the plant. It is transmitted by the Asian citrus psyllid *Diaphorina citri* and it behaves as intracellular plant pathogen and as an insect symbiont. These characteristics has made the control and eradication of *L. asiaticus* a scientific challenge with no precedents. The lack of stable culturing conditions for *L. asiaticus*, in a laboratory setting, has severely hampered progress toward understanding the physiology and adaptive strategies of this citrus pathogen. Genome analyses have revealed a drastic reduction of the genetic information encoded by the *L. asiaticus* genome, suggesting this microorganism is highly adapted to life within its host (Duan et al., 2009). One of the functional protein clusters suffering the highest negative selection were transcription factors, which represent only 2% of the total proteins encoded in the genome (Duan et al., 2009; Pagliai et al., 2014). Interestingly, the small group of transcription factors preserved in *L. asiaticus* includes the gene systematically annotated as *CLIBASIA_01510* (here named PrbP). PrbP is a member of the CarD_CdnL_TRCF superfamily (CDD cl00588). PrbP shares low sequence identity with the transcription factor CdnL in *Myxococcus xanthus* and with CarD in the *Mycobacteriaceae* family (García-Moreno et al., 2010). Members of this protein family have been linked to pathogenesis, persistence, cell viability, and resistance to both antibiotics and environmental stress (Stallings et al., 2009; Stallings and Glickman, 2011; Weiss et al., 2012).

In absence of laboratory culturing conditions for *L. asiaticus*, traditional methods for testing chemicals as antimicrobials *in vivo* cannot be applied. In this study, we explored the possibility of identifying specific ligands *in vitro*, that affect the activity of PrbP *in vivo*, as a means to disrupt gene expression, and ultimately decrease viability of *L. asiaticus* in the citrus host. Only three eukaryotes, *Spinacia oleracea, Beta vulgaris*, and *Drosophila elegans*, were found to carry PrbP homologs (74, 72, and 46% identity, respectively). The absence of a PrbP homolog in citrus renders PrbP a unique target for therapeutics. Furthermore, the use of ligands that inactivate specific pleiotropic transcription factors, enables the selective elimination of target species in heterogeneous populations. Taken together, PrbP represents an excellent therapeutic target for the design of antimicrobial strategies against *L. asiaticus*.

## Materials and methods

### Bacterial strains and growth conditions

*Escherichia coli* strains were grown at 37°C under aerobic conditions in Luria-Bertani medium (LB) (Difco) or on LB agar plates. *Escherichia coli* strains DH5α (Invitrogen, Carlslab, CA), TOP 10 (Invitrogen), and XL1-Blue (Stratagene, La Jolla, CA) were used to propagate the plasmids for protein purification, point mutations, and the two-hybrid system (the bacterial strains and plasmids used in this study are listed in Table 1). *Escherichia coli* strain BL21-Rosetta(DE3) (Novagen, Gibbstown, NJ) was used for overexpression and protein purification. When required, the medium was supplemented with ampicillin (100 μg/ml), tetracycline (10 μg/ml), kanamycin (50 μg/ml), or chloramphenicol (25 μg/ml). All antibiotics and chemicals were purchased from Sigma (St. Louis, MO).

**Table 1:** **Bacterial strains and plasmids used in this study**.

**Strain or plasmid**	**Genotype or description**	**References**
**STRAINS**		
DH5α	F−Φ80*lac*ZΔM15 Δ(*lac*ZYA-*arg*F) U169 *rec*A1 *end*A1 *hsd*R17 $\left({\text{r}}_{\text{K}}^{-},{\text{m}}_{\text{K}}^{+}\right)$ *pho*A *sup*E44 λ– *thi*-1 *gyr*A96 *rel*A1	Invitrogen
BL21-Rosetta(DE3)	F−*ompT hsdS* B(rB− mB−) *gal dcm* (DE3) pRARE	Novagen
JM109	*e14− (McrA−) recA1 endA1 gyrA96 thi-1 hsdR17 $\left({\text{r}}_{\text{K}}^{-},{\text{m}}_{\text{K}}^{+}\right)$ supE44 relA1 Δ(lac-proAB) [F′ traD36 proAB lacIq ZΔM15]*	Promega
TOP10	F− mcrA Δ(mrr-hsdRMS-mcrBC) ϕ80lacZΔM15 Δ*lac*X74 *nupG recA*1 *araD*139 Δ*(ara-leu*)7697 *galE*15 *galK*16 *rpsL*(Str^r^) *endA*1 λ−	Invitrogen
LB01	TOP10 carrying empty pB2HΔα and pB2HΔω; Amp^r^; Cm^r^	This work
LB02	TOP10 carrying pB2HΔ*α_prbP* and pB2HΔ*ω_rpoB_21–150_*; Amp^r^; Cm^r^	This work
LB03	TOP10 carrying pB2HΔ*α_ rpoB_21–150_* and pB2HΔ*ω_ prbP*; Amp^r^; Cm^r^	This work
LB04	TOP10 carrying pB2HΔα_*prbP* and pB2HΔω; Amp^r^; Cm^r^	This work
LB05	TOP10 carrying pB2HΔα and pB2HΔ*ω_rpoB_21–150_*; Amp^r^; Cm^r^	This work
*L. crescens* BT-1	Wild type *L. crescens* strain BT-1	CP003789
**PLASMIDS**		
p15TV-L	Expression vector, adds 6X His tag, TEV cleavage site, Amp^r^	EF456736
p15TV-PrbP	*prbP* gene from *L. asiaticus* cloned in the *Bse*RI site of p15TV-L	This work
pB2HΔα	pACYCDuet-1*tac* with the *E. coli* β-galactosidase fragment lacking the sequence for amino acids 11–41 (Δα) cloned in the *Bam*HI-*Nco*I site; Cm^r^	Borloo et al., 2007
pB2HΔω	pETDuet-1ΔSphIΩtac with the *E. coli* β-galactosidase fragment lacking the sequence for amino acids 789−1023 (Δω) cloned in the *Bam*HI−*Nco*I site; Amp^r^	Borloo et al., 2007
pB2HΔα_*prbP*	pB2HΔα with the *prbP* gene from *L. asiaticus* cloned in the *Not*I-*Bam*HI site; Cm^r^	This work
pB2HΔα_*rpoB_21–150_*	pB2HΔα with a portion of the *rpoB* gene (encoding residues *21–150*) from *L. asiaticus* cloned in the *Not*I-*Bam*HI site; Cm^r^	This work
pB2HΔω_*prbP*	pB2HΔω with the *prbP* gene from *L. asiaticus* cloned in the *Not*I-*Bam*HI site; Amp^r^	This work
pB2HΔω_*rpoB*	pB2HΔω with a portion of the *rpoB* gene (encoding residues 21–150) from *L. asiaticus* cloned in the *Not*I-*Bam*HI site; Amp^r^	This work

*Km^r^, Amp^r^, Cm^r^, and Tet^r^ indicate resistant to kanamycin, ampicillin, chloramphenicol, and tetracycline, respectively*.

*Liberibacter crescens* BT-1 was cultured at 25°C with moderate aeration (150 RPM), in modified BM7 media (Leonard et al., 2012) containing 1% Brain Heart Infusion (Difco Laboratories, Detroit, MI), 15% Fetal Bovine Serum (Sigma, St. Louis, MO), 30% TMN-FH insect medium (Sigma), α-Ketoglutaric acid (2 mg/ml), ACES (10 mg/ml), and potassium hydroxide (3.75 mg/ml), at pH 6.9. For determinations of the antimicrobial activity of metronidazole, dimetridazole, ronidazole, and tolfenamic acid, each chemical was added to the liquid media at increasing concentrations (1–500 μM). The optical density was determined at 600 nm every 24 h during 5 days.

### DNA manipulations and gene cloning

Standard methods were used for chromosomal DNA isolation, restriction enzyme digestion, agarose gel electrophoresis, ligation, and transformation (Pagliai et al., 2010). Plasmids were isolated using the QIAprep® Spin Miniprep Kit (Qiagen, Valencia, CA), and PCR products were purified using Qiaquick® Purification Kits (Qiagen). All of the primers used in this study are described in Table 2. For protein expression and purification, the *prbP* (*CLIBASIA_01510*) gene was amplified by PCR, using total DNA extractions from *L. asiaticus* infected tissue, and cloned into the p15TV-L plasmid as described previously (Pagliai et al., 2010).

**Table 2:** **Oligonucleotides used in this study**.

**Primer**	**Sequence (5′ → 3′)**
**qRT-PCR**	
CLIBASIA_01510-Fw	CTGCCCATGGAGTAGGAACTATTAC
CLIBASIA_01510-Rv	ATCTTGTCCTTGTCAAATGCAATAA
CLIBASIA_00120-Fw	TGGAGGTGTAAAAGTTGCCAAA
CLIBASIA_00120-Rv	CCAACGAAAAGATCAGATATTCCTCTA
CLIBASIA_r05785-Fw	TCGAGCGCGTATGCGAATACG
CLIBASIA_r05785-Rv	GCGTTATCCCGTAGAAAAAGGTAG
CLIBASIA_00130-Fw	TCGGGATCTAAACTTCCTGGT
CLIBASIA_00130-Rv	ATAGCCCCCATATCTTGCATC
CLIBASIA_00325-Fw	TATCCCAATGTGCTGGTCAA
CLIBASIA_00325-Rv	GACCCGTTGCATAAGCATTT
CLIBASIA_00735-Fw	CGATGGAGCCAAATCAGACT
CLIBASIA_00735-Rv	GGACCTACAATCTGCGAACC
COX-Fw	GTATGCCACGTCGCATTCCAGA
COX-Rv	GCCAAAACTGCTAAGGGCATTC
18S-Fw	GCTTAGGCCAAGGAAGTTTG
18S-Rv	TCTATCCCCATCACGATGAA
**DNASE I FOOTPRINT**	
CLIB_00130_Fw_Fam	CTGATGGTCCGTTTGCTTCT
CLIB_00130_Rv_Vic	TGCAGAACCCGACTCTATCTG
**EMSA**	
EMSA_CLIB_00130_Ext_Fw	CTGTTTTCTTCGAGGTTGGTG
EMSA_CLIB_00130_Ext_Rv	CCGCATTAAACGCCTTACAA
EMSA_CLIB_00130_Fw	CTGATGGTCCGTTTGCTTCT
EMSA_CLIB_00130_Rv_Bio	TGCAGAACCCGACTCTATCTG
EMSA_Cold_CLIB_00130_Rv	TGCAGAACCCGACTCTATCTG
EMSA_CLIB_01510_Fw_Bio	TCTAAACCCTTGGCGCATC
EMSA_CLIB_01510_Rv	TGAAAAGTATTTCTCCCCTAATCC
EMSA_Cold_CLIB_01510_Fw	TCTAAACCCTTGGCGCATC
EMSA_CLIB_RadA_Ext_Fw	GTCTTGTGGCGTTTCACATC
EMSA_CLIB_RadA_Ext_Rv	CAGAAGCAAGGGCTACATCA
EMSA_CLIB_RadA_Fw	TCATCTTGCTTGTGCTGATG
EMSA_CLIB_RadA_Rv_Bio	GGCTATCAGACTATCGCGTGT
EMSA_Cold_CLIB_RadA_Rv	GGCTATCAGACTATCGCGTGT
EMSA_CLIB_DnaK_Ext_Fw	TGTCTCCGTATCAACTGCAA
EMSA_CLIB_DnaK_Ext_Rv	ACTGTTTTCCCTGTGCTTCG
EMSA_CLIB_DnaK_Fw	AATTTTTCCTTGCAATCAAGC
EMSA_CLIB_DnaK_Rv_Bio	TCCATAATAGCAACGCATGAA
EMSA_CLIB_RpsJ_Ext_Fw	TGCTCCTGGTTCGATTCAA
EMSA_CLIB_RpsJ_Ext_Rv	CCGCATTTCCAATTGATCTC
EMSA_CLIB_RpsJ_Fw	CGATGGAGCCAAATCAGACT
EMSA_CLIB_RpsJ_Rv_Bio	GGACCTACAATCTGCGAACC
EMSA_CLIB_r05785_Fw	AGAAGAAAGGGAGACGTGGA
EMSA_CLIB_r05785_Rv	CCATGCGTTATCCCGTAGAA
**SITE DIRECTED MUTAGENESIS**	
rplKM1_Fw	GTGTTTGTTTTATATAGTCGGTTGGTTGTTTTTTAG
rplKM1_Rv	CTAAAAAACAACCAACCGACTATATAAAACAAACAC
rplKM2_Fw	GTGTTTGTTTTATATAGTATATTGGTTGTTTTTTAG
rplKM2_Rv	CTAAAAAACAACCAATATACTATATAAAACAAACAC
rplKM3_Fw	GTTTTATATAGTAGGCCGGTTGTTTTTTAGAAAGG
rplKM3_Rv	CCTTTCTAAAAAACAACCGGCCTACTATATAAAAC
rplKM4_Fw	GTTTTATATAGTAGGTTAATTGTTTTTTAGAAAGGCTAGG
rplKM4_Rv	CCTAGCCTTTCTAAAAAACAATTAACCTACTATATAAAAC
rplKM5_Fw	GTTTTATATAGTAGGTTGGCCGTTTTTTAGAAAGGCTAGGG
rplKM5_Rv	CCCTAGCCTTTCTAAAAAACGGCCAACCTACTATATAAAAC
rplKM6_Fw	GTTTTATATAGTAGGTTGGTTGCCTTTTAGAAAGGCTAGGG
rplKM6_Rv	CCCTAGCCTTTCTAAAAGGCAACCAACCTACTATATAAAAC
rplKM7_Fw	GTTTTATATAGTAGGTTGGTTGTTCCTTAGAAAGGCTAGGGATGGC
rplKM7_Rv	GCCATCCCTAGCCTTTCTAAGGAACAACCAACCTACTATATAAAAC
rplKM8_Fw	GTGTTTGTTTTATATAATAGGTTGGTTGTTTTTTAG
rplKM8_Rv	CTAAAAAACAACCAACCTATTATATAAAACAAACAC
rplKM9_Fw	GTAGGTTGGTTGTTTTTTATAAAGGCTAGGGATGGC
rplKM9_Rv	GCCATCCCTAGCCTTTATAAAAAACAACCAACCTAC
rplKM10_Fw	GGTTGGTTGTTTTTTAGCCAGGCTAGGGATGGCAAAG
rplKM10_Rv	CTTTGCCATCCCTAGCCTGGCTAAAAAACAACCAACC
rplKM11_Fw	GGTTGGTTGTTTTTTAGACCGGCTAGGGATGGCAAAG
rplKM11_Rv	CTTTGCCATCCCTAGCCGGTCTAAAAAACAACCAACC
rplKM12_Fw	GGTTGGTTGTTTTTTAGAAATGCTAGGGATGGCAAAG
rplKM12_Rv	CTTTGCCATCCCTAGCATTTCTAAAAAACAACCAACC
**TWO-HYBRID CONSTRUCTS**	
*prbP*-Fw	tattgcggccgcATGACATTCCAACAGAAAAGAGATG
*prbP*-Rv	tatatggatccTGCGGCTTTATCTTGATTTTCGCTT
*rpoB*_Ext-Fw	CGTTGTGTTCAATGGTCTCG
*rpoB*_Ext-Rv	GGAACCTTGCGACGTCTATC
*rpoB*-Fw	tattgcggccgcCCTGAGATAATTGACATACCTGATCT
*rpoB*-Rv	tatatggatccCTGAATACCC TTAATAACGA AAGTTCC
**OVEREXPRESSION AND PROTEIN PURIFICATION IN p15TV-L**	
CLIBASIA_01510_LIC-Fw	TTGTATTTCCAGGGC ATGACATTCCAACAGAAAAGAGATG
CLIBASIA_01510_LIC-Rv	CAAGCTTCGTCATCA CTATGCGGCTTTATCTTGATTTTC
CLIBASIA_01510_Ext-Fw	GAGTGTGCGTTTGTTTGAAAAG
CLIBASIA_01510_Ext-Rv	CCCACGCGATCTTATCTGAC

*Restriction sites are underlined*.

### Two-hybrid system

The two-hybrid system previously described by Borloo et al. (2007) was used (Borloo et al., 2007). Proteins of interest were fused to complementing β-galactosidase truncations (Δα and Δω), where the resulting level of complemented β-galactosidase activity corresponds directly to the level of interaction between the proteins. Proteins of interests were cloned into vectors pB2HΔα and pB2HΔω using the *Not*I and *Bam*HI restriction sites. All subcloning steps were performed in *E. coli* XL-1 Blue (Stratagene, La Jolla, CA). Fusion proteins in pB2HΔα and pB2HΔω were transformed by heat shock and subsequently co-expressed in *E. coli* TOP10. Empty vectors were used as a control to determine baseline activity. The minimal inhibitory concentration (MIC) was determined for each chemical in *E. coli* (Table 3). The highest concentration that did not adversely affect the base levels in the controls was used for subsequent *in vivo* assays.

**Table 3:** **Small molecules used in this study**.

**Chemical**	**ΔTm^a^ (°C)**	**MIC^b^ (μM)**	**Concentration^c^ tested (μM)**	**β-galactosidase activity^d^ (% decrease)**
Berberine	1.5	100	50	5.1 ± 0.6
Acetazolamide	1.7	>200	50	3.7 ± 0.4
Metronidazole	1.8	100	50	53.7 ± 10
Gramine	−2.5	100	40	15.3 ± 0.3
Cotine	2.1	50	10	5.2 ± 0.8
Tolfenamic acid	−4.2	100	25	17.5 ± 5.3
Folic acid	−3.5	>400	200	2.2 ± 0.2
Menadione	−2.5	50	15	9.8 ± 2.3
2-Methyl-4(5)-nitroimidazole	ND	>200	100	17.5 ± 0.2
Ornidazole	0.5	50	10	11.6 ± 2.4
Dimetridazole	ND	50	10	25.8 ± 4.6
1,2-Dimethylimidazole	ND	>200	200	37.6 ± 6.1
Ronidazole	ND	10	1	48.2 ± 3.9

*The effect of small molecules on the thermal stability of PrbP (ΔTm) and on interactions between PrbP and RpoB (β-galactosidase activity)*.

a *ΔTm was calculated as the difference in the transition temperature of PrbP in the absence (PrbP Tm = 37.3°C) and presence of each chemical. The results are the average of duplicates*.

b *MIC: Minimal Inhibitory Concentration for the E. coli TOP10 reporter strain*.

c *Concentration tested: Concentration used in the bacterial two-hybrid system*.

d *β-galactosidase activity (expressed as arbitrary units, AU) as a result of pB2HΔα-prbP and pB2HΔω-rpoB_21–150_ interaction is expressed as a decrease in the activity in the presence of chemicals, compared to the control without chemicals after 240 min (OD = 0.8). The assay was performed a minimum of three times, each in triplicates*.

### β-galactosidase assays

*Escherichia coli* cells were grown at 37°C in LB medium until reaching OD600 of 0.8 (late exponential phase). Cells were collected and lysed in Z-buffer (60 mM Na_2_HPO_4_, 40 mM NaH_2_PO_4_, 10 mM KCl, 1 mM MgSO_4_, 50 mM β-mercaptoethanol; Miller, 1972). β-galactosidase activity was assayed by following the catalytic hydrolysis of chlorophenol red-β-D-galactopyranoside (Sigma-Aldrich). The absorbance at 570 nm was read continuously using a Synergy HT 96-well plate reader (BioTek, Winooski, VT). β-galactosidase activity, expressed as arbitrary units (AU), was calculated using the slope of absorbance curve normalized with the initial cell density. Preliminary β-galactosidase assays were performed with strains LB01 and LB02, in the absence and presence (0–1 mM) of each chemical to determine baseline activity. Each assay was performed in triplicates.

### Protein purification

Protein purification was performed as previously described (Lorca et al., 2007). Briefly, PrbP was cloned into vector p15TV-L. The His-tagged fusion protein was then overexpressed in *E. coli* BL21-Rosetta(DE3) (Novagen). Cells were grown in LB broth at 37°C, to an OD600 of 0.6. Expression was induced with 0.5 mM isopropyl -thio-β-D-galactopyranoside (IPTG). After induction, the cells were incubated at 17°C for 16 h. The cells were harvested and resuspended in binding buffer (500 mM NaCl, 5% glycerol, 50 mM HEPES, 5 mM imidazole, pH 7.5) with Roche EDTA-free protease inhibitor cocktail (Roche Applied Science, Germany). Phenylmethylsulfonyl fluoride (0.5 mM) and Tris(2-carboxyethyl)phosphine hydrochloride (0.5 mM) were added to the cells immediately before lysing. Cells were lysed using a french press. The lysates were clarified by centrifugation (30 min at 17,000 × g) and applied to a metal chelate affinity column charged with nickel. The column was washed extensively with binding buffer containing 20 mM imidazole, and the proteins were subsequently eluted from the column with elution buffer (binding buffer containing 250 mM imidazole). The purified proteins were dialyzed against 10 mM HEPES (pH 7.5), 500 mM NaCl, 2.5% glycerol, 0.5 mM TCEP, and stored at −80°C. The identity of the purified proteins was confirmed by Mass Spectrometry (as a service in the Interdisciplinary Center for Biotechnology Research, University of Florida) from protein bands isolated from SDS-PAGE gels.

### Electrophoretic mobility shift assays (EMSA)

Gel shift assays for PrbP were performed using aliquots of protein purified and concentrated according to the procedures described above. Fragments of the *rplK, prbP, rpsJ, dnaK*, and *radA* promoter regions were generated by PCR using pre-labeled 5′-biotin primers (Table 2). PCR products were purified using QIAquick spin columns (Qiagen). EMSA reaction mixtures (20 μL) contained 1 ng of 5′ biotin-labeled DNA probe, 12.5 ng/μL of both Poly(dI-dC) and Poly(dA-dT) nonspecific competitor DNAs, 10 mM Hepes pH 7.5, 150 mM NaCl, 5% glycerol, purified PrbP protein (0–7 μM), and ligand (0–2 mM)as indicated. Competition assays were carried out using unlabeled fragments of the promoter regions generated by PCR. After incubation at 37°C for 20 min, samples were separated on 6% acrylamide-bisacrylamide non-denaturing gels, in ice cold Tris borate-EDTA buffer, pH 8.3 (TBE). Electrophoresis was performed on ice at 100 V for 2 h. DNA was transferred to a Hybond-N^+^ membrane (GE Healthcare, Pittsburgh, PA) with a Semi-Dry electroblotter (Fisher Scientific, Pittsburgh, PA) at 250 mA for 45 min. Transferred DNA was cross-linked to the membrane using a Spectrolinker XL-1000 UV cross-linker equipped with 312 nm UV bulbs. Biotin labeled DNA was detected using a Phototope-Star Detection Kit (New England Biolabs, Ipswich, MA). Membranes were exposed to Kodak X-ray film.

### DNase I footprinting

Protection assays were performed on both minus and plus strands using 5′-6FAM or 5′-VIC labeled probes that were generated by PCR (primers described in Table 2). 1.5 μg of labeled *P*_*rplK*_ probe was combined with 40 μM PrbP, 0.5 mM CaCl_2_, 2.5 mM MgCl_2_, and 0.025 U of DNase I (New England Biolabs) in a 200 μl reaction. After incubation at 37°C for 20 min, the reaction was stopped by the addition of 50 mM EDTA, pH 8.0. As a control, a digestion reaction was performed under the same conditions without PrbP. The digested DNA and the sequencing reaction products were analyzed at the Plant and Microbe Genomics facility, Ohio State University, Columbus, using a 3730 DNA analyzer. The protected regions were identified using GeneMapper software (Life Technology), as previously described (Zianni et al., 2006).

### Small molecule screening by differential scanning fluorimetry

Purified PrbP protein was screened against the Prestwick chemical library of 1152 compounds (Prestwick Chemical, France) using differential scanning fluorimetry as previously described (Vedadi et al., 2006; Niesen et al., 2007; Wrench et al., 2013). Briefly, purified PrbP protein was diluted to a final concentration of 20 μM in 100 mM HEPES, pH 7.5, 150 mM NaCl. 20 μl aliquots of a protein solution containing the chemical compounds were placed in duplicate, into 96-well plates (Bio-Rad) and heated from 25 to 80°C, at the rate of 1°C per min. A real time PCR device (iCycler IQ™, Bio-Rad) was used to monitor protein unfolding by an increase in the fluorescence of the fluorophore SYPRO Orange (Invitrogen). Fluorescence intensities were plotted against temperature for each sample well, and transition curves were fitted with the Boltzmann equation using Origin 8 software (Northampton, MA). The midpoint of each transition was calculated and compared with the midpoint calculated for the reference sample. If the difference between the midpoints was greater than 2.0°C, the corresponding compound was considered to be a “hit.”

### Analyses of chemicals on infected leaves

Leaves were collected from HLB-symptomatic Valencia Orange (*C. sinensis*) trees, maintained at the University of Florida main campus. All leaves used in this study were collected from new flushes on highly symptomatic branches. Prior to treatment all solutions were autoclaved or filter sterilized. 100 μM stocks of the chemical were prepared in 10 mM Tris pH 8.0. A solution of 10 mM Tris pH 8.0 was used for the controls. A scalpel was used to harvest leaves from the tree, with a horizontal cut at the base of the petiole. Each leaf was immediately suspended in 8 ml of treatment solution (with or without chemicals). Leaves were supported in a vertical position throughout the incubation period, with only the lower inch of the petiole submerged in solution (with or without chemical). Steady air flow was maintained over the leaf blades throughout the incubation period, to facilitate transpiration and the adsorption of each solution. Each treatment group consisted of 12 leaves that were processed after 24 h of incubation (with or without chemical).

For each treatment group, biological quadruplicates were prepared from the twelve leaves. The leaf midribs and petioles were collected and incubated overnight at 4°C in RNA*later* solution (Life Technologies, Grand Island, NY). Following treatment with RNA*later*, samples were rinsed twice with RNase free water and immediately frozen in liquid nitrogen. Samples were then freeze dried over a period of 3 days and homogenized using a GenoGrinder 2000. Homogenized samples were stored at −80°C.

### RNA purification and cDNA synthesis

Plant and bacterial RNA was extracted from 75 mg of homogenized tissue. Extractions were carried out using the Isolate II RNA Plant Kit (Bioline, London, UK) with lysis buffer RLY. Zirconia beads (0.1 mm) were added to each sample during lysis, to aid with the disruption of bacterial cells. Purified RNA was eluted with 60 μl of RNase/DNase-free water, and subsequently treated with TURBO DNA-*free* DNase (Thermo Scientific, Wilmington, DE) to eliminate trace amounts of DNA. Purified RNA samples were quantified using a NanoDrop ND 1000 (Thermo Scientific, Wilmington, DE) and stored at −80°C. cDNA was synthesized using the iScript cDNA Synthesis Kit (Bio-Rad, Hercules, CA) with random hexamer primers and 0.5 μg of RNA. cDNA products were diluted 50% with DNase/RNase-free water and stored at −80°C.

### Real-time quantitative PCR (qRT-PCR) analysis

qRT-PCR was carried out in a iCycler IQ apparatus (Bio-Rad) using Platinum SYBR Green qPCR SuperMix for iCycler (Life Technologies) in accordance with the manufacturer's recommended protocol. Reactions were carried out using 2 μl of cDNA, in a total reaction volume of 26 μl. The genes measured for *L. asiaticus* included *prbP* (*CLIBASIA_01510*), *L25* (*CLIBASIA_01515*), *rplK* (*CLIBASIA_00130*), *rpsJ* (CLIBASIA_00735), *gyrA* (*CLIBASIA_00325*), and 16S rRNA (*CLIBASIA_r05785)*. The mRNA levels of *rplK, CLIBASIA_r05785, rpsJ and prbP* genes were normalized to the abundance of the plant genes *cox2* and 18S rRNA. The expression of each plant control gene was previously examined in absence and presence of tolfenamic acid (data not shown). Quantitative reverse transcription-PCR primers are described in detail in Table 2.

### Evaluation of phytotoxicity on sweet orange seedlings

Twelve-month old seedlings were randomly divided into ten groups of 8 seedlings. Tolfenamic acid was prepared in 10 mM tris buffer, and applied at concentrations of 1, 10, 100, 1000, and 10,000 μM by root soaking (with 100 ml), foliar spray (to saturation), or root soaking and foliar spray combined. A control group was also treated with 10 mM tris buffer only. Each treatment was applied twice at 2 week intervals. A non-treated control group was also maintained throughout the duration of the experiment. Seedlings were monitored for symptoms of phytotoxicity during a period of 12 months following treatment. No phytotoxic effects were observed following treatment with tolfenamic acid (up to 10 mM). Tolfenamic acid had no effect on the transcription of Cox2 or 18S rRNA (data not shown).

### Evaluation of antimicrobial efficacy of tolfenamic acid against *L. asiaticus*

*Citrus sinensis* “Valencia” inoculation: Twelve-month old seedlings were graft inoculated with budwood collected from HLB-infected trees. Prior to grafting, the source of the infected tissue was analyzed by PCR to confirm the presence of viable *L. asiaticus*. PCR confirmation was carried out using primers for 16S rRNA (CLIBASIA_r05781) and *gyrA* (CLIBASIA_00325) genes (see Table 2 for primer sequences). Inoculated plants were kept in a secure greenhouse approved by the USDA Animal and Plant Health Inspection Service (APHIS). The plants were watered in accordance with the standard watering schedule for the commercial citrus industry. Citrus fertilizer (Sunniland Citrus 6-4-6, N-P-K) was applied every 2 months as instructed by the manufacturer. Two months after grafting, each plant was tested for HLB by PCR as described above.

Seedlings treatment study: After 9 months of testing positive for HLB, the highly symptomatic, infected sweet orange seedlings (described above) were randomly divided into two groups. 100 μM tolfenamic acid (TA) was applied to one group of infected seedlings as a foliar spray (to saturation), and by root soaking (with 100 ml of 100 μM TA). The buffer vehicle (10 mM tris) was applied to the control group as a foliar spray (to saturation), and by root soaking (with 100 ml of 10 mM tris). Each treatment was applied twice, at 2 week intervals. Evaluation of HLB symptoms (leaf yellowing and mottling) and *L. asiaticus* transcriptional activity (as a measure of viability) was performed as described above.

### Statistical analyses

qRT-PCR statistical significance was assessed using a two-tail *P*-value, calculated with the Mann–Whitney nonparametric test.

## Results

### PrbP binds specifically to the promoter region of *rplK*

**Figure 1:**
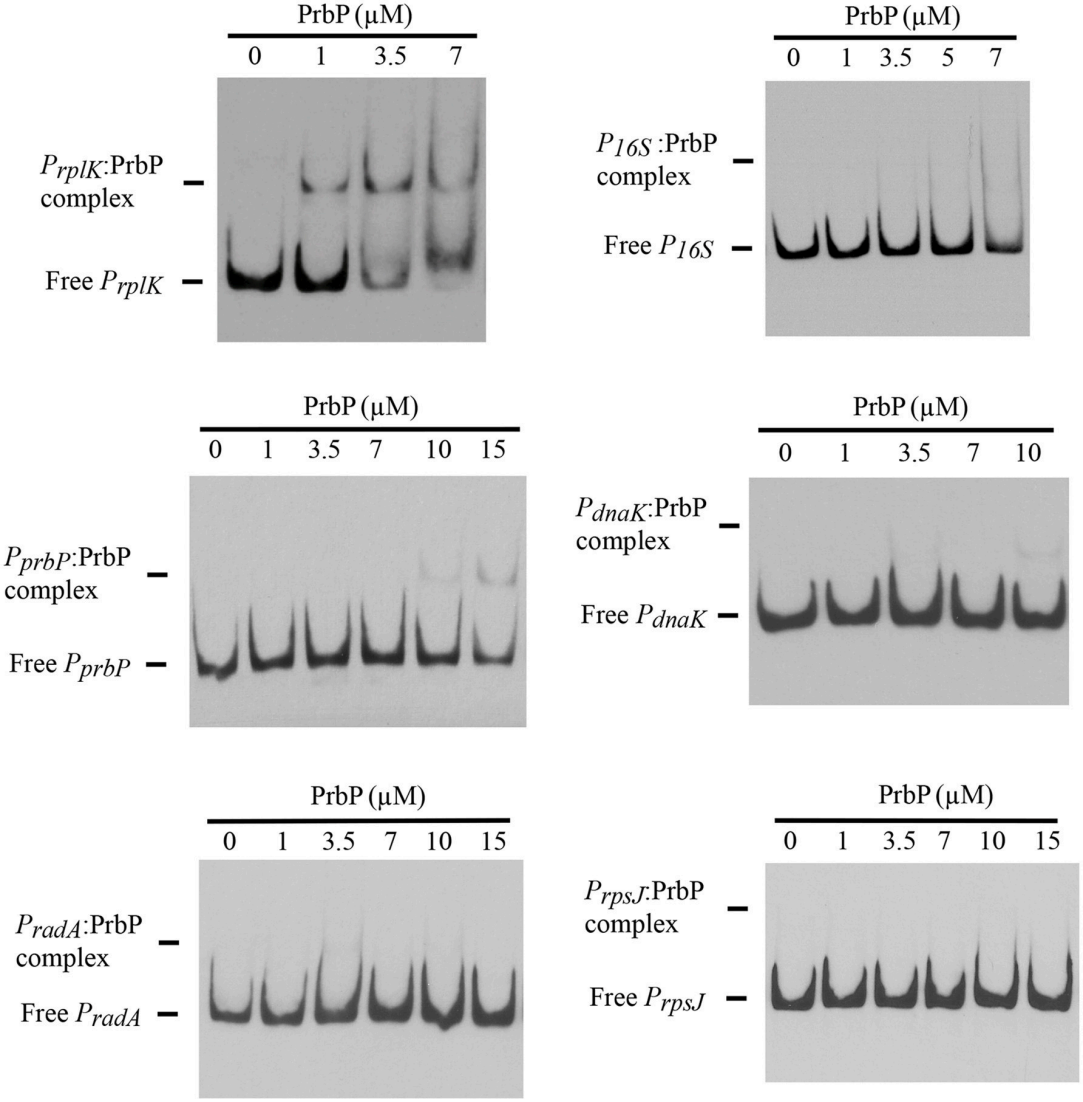
**DNA binding assays with PrbP**. EMSAs were conducted with 1 ng of biotin labeled probes *P*_*rplK*_, *P*_**16*S*_, *P*_*rpsJ*_, *P*_*prbP*_, *P*_*dnaK*_, or *P*_*radA*_ and increasing concentrations (0–15 μM) of PrbP, as indicated on top of each panel. No protein was added to the first lane of each gel.

**Figure 2:**
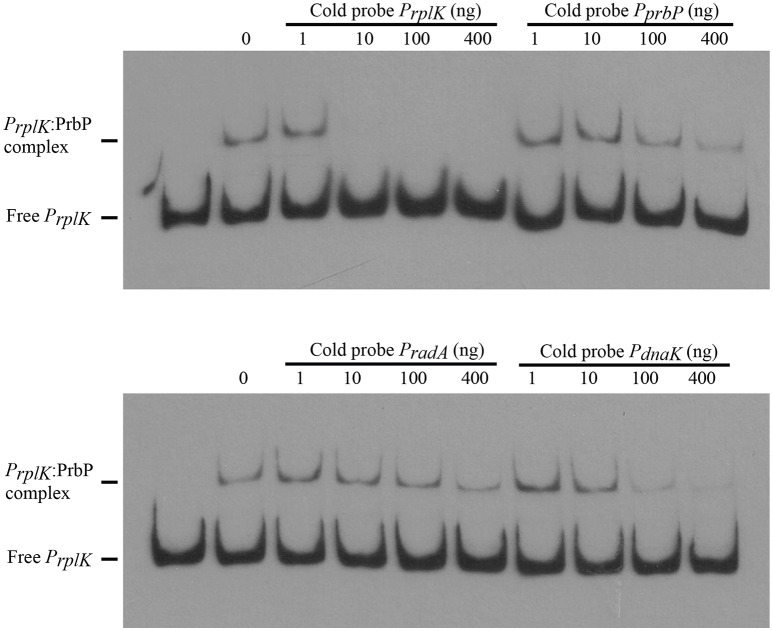
**PrbP binds specifically to ***rplK*** promoter region**. For competition experiments, a biotin labeled *P*_*rplK*_ probe was incubated with 2.5 μM PrbP and mixed with increasing concentrations (1–400 ng) of an unlabeled, double stranded probe (*P*_*rplK*_, *P*_*prbP*,_ *P*_*radA*__,_ or *P*_*dnaK*_), as indicated above each panel.

**Figure 3:**
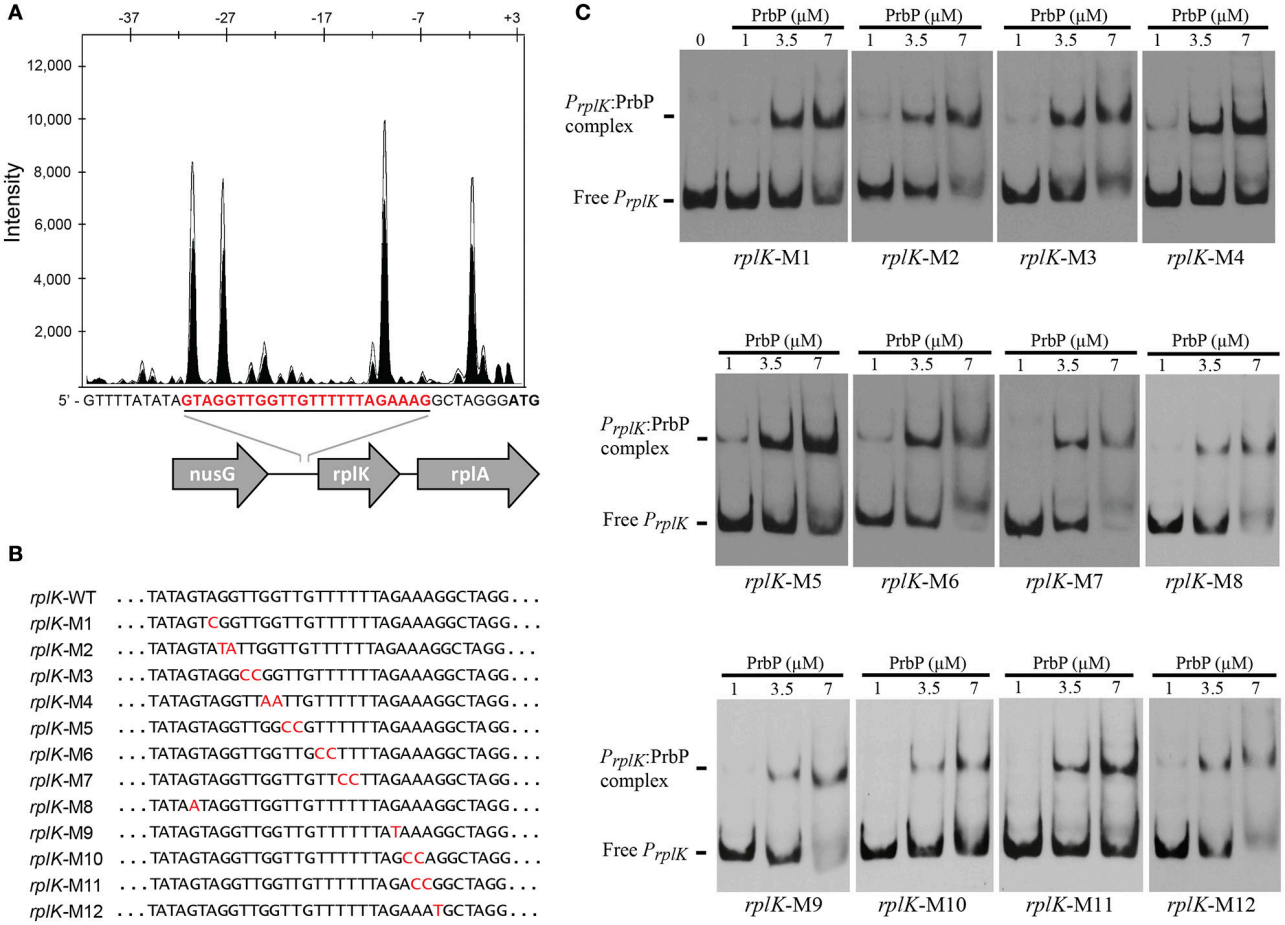
**Identification of PrbP binding site in ***rplK*** promotor (***P***_***rplK***_)**. **(A)** DNAse I footprint assays identified a protected site (red) located 31 bp from the *rplK* translation start site. The electropherogram shows a fragment of the digested probe in absence (white) or presence (black) of PrbP, highlighting the protected region. **(B)** Site directed mutagenesis of the *rplK* promoter region. Mutated residues are indicated (in red) for each of the *rplK* mutants (*rplK*-M1–*rplK*-M12). **(C)** EMSA assays were conducted using 1 ng of each mutant *P*_*rplK*_ probe (*rplK*-M1–*rplK*-M12) with increasing concentrations (0–7 μM) of PrbP_,_ as indicated on top of each panel. The amount of free *P*_*rplK*_ was used to determine the binding efficiency of PrbP with each mutant probe.

Previous studies in PrbP homologs have found that these proteins may bind non-specifically to the promoter regions of the *rrnA*, and *rpsH* genes (Gulten and Sacchettini, 2013; Srivastava et al., 2013). In this study, we examined the DNA binding properties of PrbP in *L. asiaticus*, using the promoter region of genes encoding the ribosomal proteins *rplK* (CLIBASIA_00130; *P*_*rplK*_) and *rpsJ* (CLIBASIA_00735; *P*_*rpsJ*_), as well as the promoter regions of 16S rRNA (CLIBASIA_r05785; *P*_16*S*_), *prbP* (CLIBASIA_01510; *P*_*prbP*_), *dnaK* (CLIBASIA_02620; *P*_*dnaK*_), and *radA* (CLIBASIA_01095; *P*_*radA*_). We found that PrbP was able to bind *P*_*rplK*_ at 3.5 μM and *P*_16*S*_ at 5 μM, while binding to *P*_*rpsJ*_, *P*_*prbP*_, *P*_*dnaK*_, and *P*_*radA*_ was only observed with higher concentrations (10–15 μM) of PrbP (Figure 1). Competition assays were used to confirm the specificity of the PrbP:*P*_*rplK*_ interaction, where a 10-fold excess of unlabeled *P*_*rplK*_ was found to completely out-compete the labeled *P*_*rplK*_ fragment (Figure 2). At higher concentrations (100–400-fold excess), *P*_*dnaK*_ was found to compete with the labeled *P*_*rplK*_ fragment, but to a lesser extent than observed with unlabeled *P*_*rplK*_. The addition of excess (up to 400-fold) unlabeled *P*_*radA*_ or unlabeled *P*_*prbP*_ had no effect (Figure 2). These results indicate that PrbP binds to a specific sequence in the promoter region of *rplK*, with higher affinity. DNase I footprinting was used to identify the DNA binding site for PrbP in the promoter region of *rplK* (Figure 3A). The 24 nucleotide protected site (GTAGGTTGGTTGTTTTTTAGAAAG), is located 31 bp from the translational start codon, on the plus strand. The critical DNA contact residues were identified by EMSA analysis, following site directed mutagenesis of the *rplK* promoter region (Figures 3B,C). Of the twelve mutant EMSA probes tested, only *rplK*-M1, *rplK*-M4, *rplK*-M5, *rplK*-M10, and *rplK*-M11 resulted in decreased binding to PrbP (Figure 3C). These results indicate that nnAnnnnGGTTnnnnnnnnnAAAn is the PrbP recognition sequence for this promoter. These results confirm that *L. asiaticus* PrbP interacts with a specific sequence in the promoter region.

### *Liberibacter asiaticus* PrbP interacts with the β-subunit of the RNA polymerase

**Figure 4:**
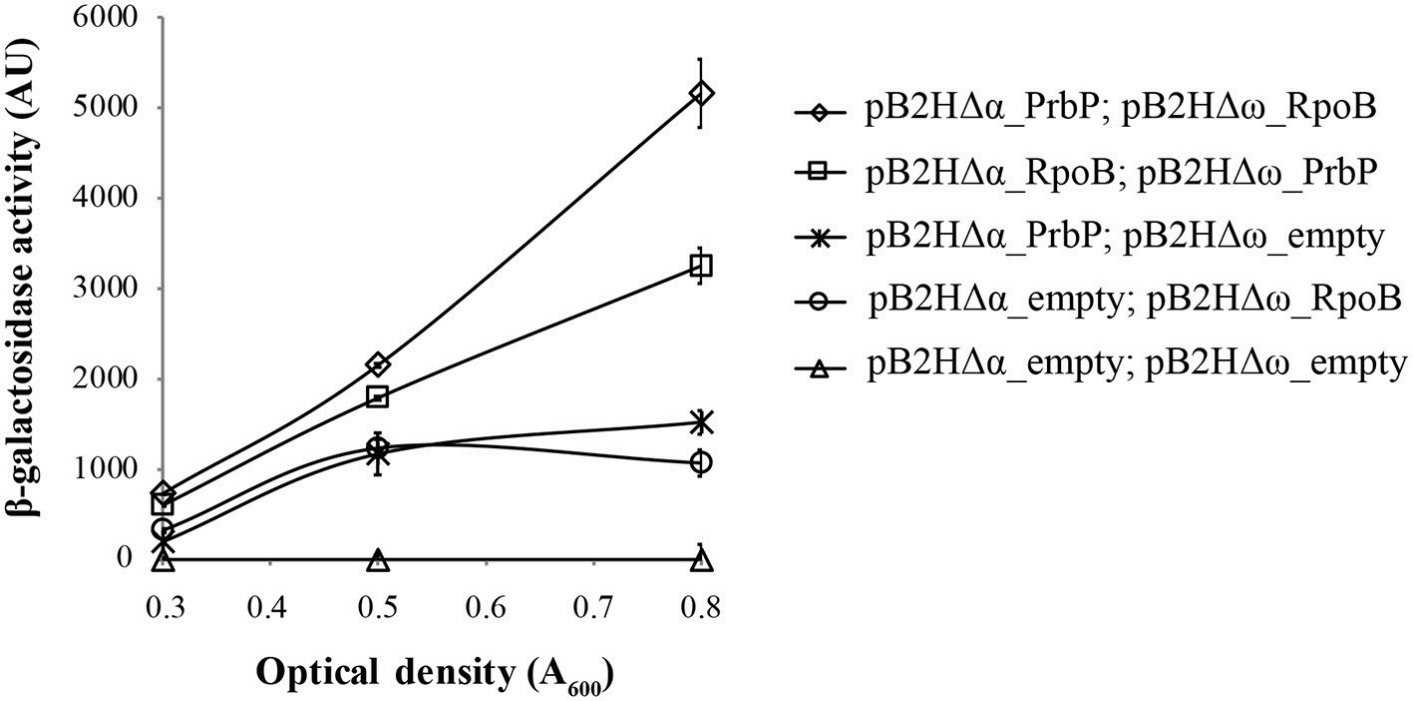
**Analysis of PrbP interactions with RpoB**. β-galactosidase activity was performed using *E. coli* TOP10 derivatives as reporter strains, transformed with the following plasmid combinations: LB01 **(**Δ**)** carrying the empty pB2HΔα and pB2HΔω plasmids; LB02 **(♢)** carrying pB2HΔα*_prbP* and pB2HΔω*_rpoB*_**21–150**_); LB03 **(□)** carrying pB2HΔα*_rpoB*_**21–150**_ and pB2HΔω*_prbP*; LB04 (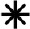) carrying pB2HΔα*_prbP* and the empty pB2HΔω; and LB05 **(O)** carrying the empty pB2HΔα and pB2HΔω*_rpoB*_**21–150**_. Assays of β-galactosidase activity are expressed in arbitrary units (AU), and were performed at different growth phases (as indicated by optical density, A_600_), in triplicates. The β-galactosidase activity for each strain was quantified after subtraction of the baseline activity.

A bacterial two-hybrid system was used to investigate potential interactions between PrbP and the β-subunit of the RNAP. Plasmids pB2HΔα and pB2HΔω were used to create fusions of the *L. asiaticus* genes *CLIBASIA_01510* (*prbP*) and *CLIBASIA_00110*_**21–150**_ (*rpoB*, residues 21–150), to the β-galactosidase truncations Δα and Δω as described by Borloo et al. (2007). Protein-protein interactions were followed by β-galactosidase activity. High levels of β-galactosidase activity were observed in strains LB02 (carrying pB2HΔα*-prbP* and pB2HΔω*-rpoB*_**21–150**_) and LB03 (carrying pB2HΔα*-rpoB*_**21–150**_ and pB2HΔω*-prbP*) (5823 ± 345 AU and 3252 ± 217AU, respectively), when compared to the control strains (Figure 4). These results confirm that *L. asiaticus* PrbP interacts with the RNAP.

### Identification of small molecules that bind PrbP

Our approach to study the biological role of PrbP was to identify small molecules that bind and/or interact with the protein to regulate its activity. To this end, the *L. asiaticus prbP* gene was cloned into vector p15TV-L, and subsequently overexpressed in *E. coli* BL21. The purified protein was screened against the Prestwick chemical library of small molecules, by differential scanning fluorimetry (Vedadi et al., 2006; Pagliai et al., 2010; Wrench et al., 2013). The midpoint transition temperature of PrbP was determined to be 37.3 ± 0.5°C. From the 1200 small molecules examined in the screening, 8 compounds were found to induce a shift in the midpoint transition temperature (Δ*Tm*) of PrbP. Metronidazole, cotine, gramine, menadione, folic acid, and tolfenamic acid were found to have the strongest effect, inducing a *Tm* shift of 1.8°, 2.1°, −2.5°, −2.5°, −3.5°, and −4.5°C, respectively (Table 3). Berberine chloride and acetazolamide were found to interact with PrbP to a lesser degree, with a Δ*Tm* of 1.5° and 1.7°C, respectively.

### Small molecules modulate PrbP interactions with the RNA polymerase

A bacterial two-hybrid system was used to further investigate the interactions between PrbP and RNAP, in the absence and presence of each chemical. The MIC was determined for each chemical in *E. coli* (Table 3). Preliminary β-galactosidase assays were performed using strains LB01 and LB02, in absence and presence (1 μM−1 mM) of each chemical to determine baseline activity. The highest concentration that did not adversely affect the base levels in the controls was used for subsequent *in vivo* assays (Table 3). Metronidazole showed the strongest effect, decreasing PrbP/RpoB interactions by 53.7%, while berberine, acetazolamide, gramine, cotine, menadione, folic acid, and tolfenamic acid had a lesser effect, decreasing the interaction by 5.1, 3.7, 15.3, 5.2, 9.8, 2.2, and 17.5%, respectively (Table 3).

Consequently, we identified five compounds with chemical scaffolds similar to metronidazole (dimetridazole, 2-methyl-4(5)-nitroimidazole, 1,2-dimethylimidazol, ornidazole, and ronidazole) and their effect was tested on the interaction between PrbP and RpoB. The inhibition values obtained were low for ornidazole (11.6%), 2-methyl-4(5)-nitroimidazole (17.5%), and dimetridazole (25.8%). When tested at higher concentrations (200 μM), 1,2-dimethylimidazole was found to decrease PrbP/RpoB interactions by 37.6%, however, no effect was observed at lower concentrations (<50 μM). Conversely, a lower concentration (1 μM) of ronidazole was found to significantly decrease (48%) PrbP/RpoB interactions (Table 3).

### Small molecules disrupt interactions between PrbP and DNA

**Figure 5:**
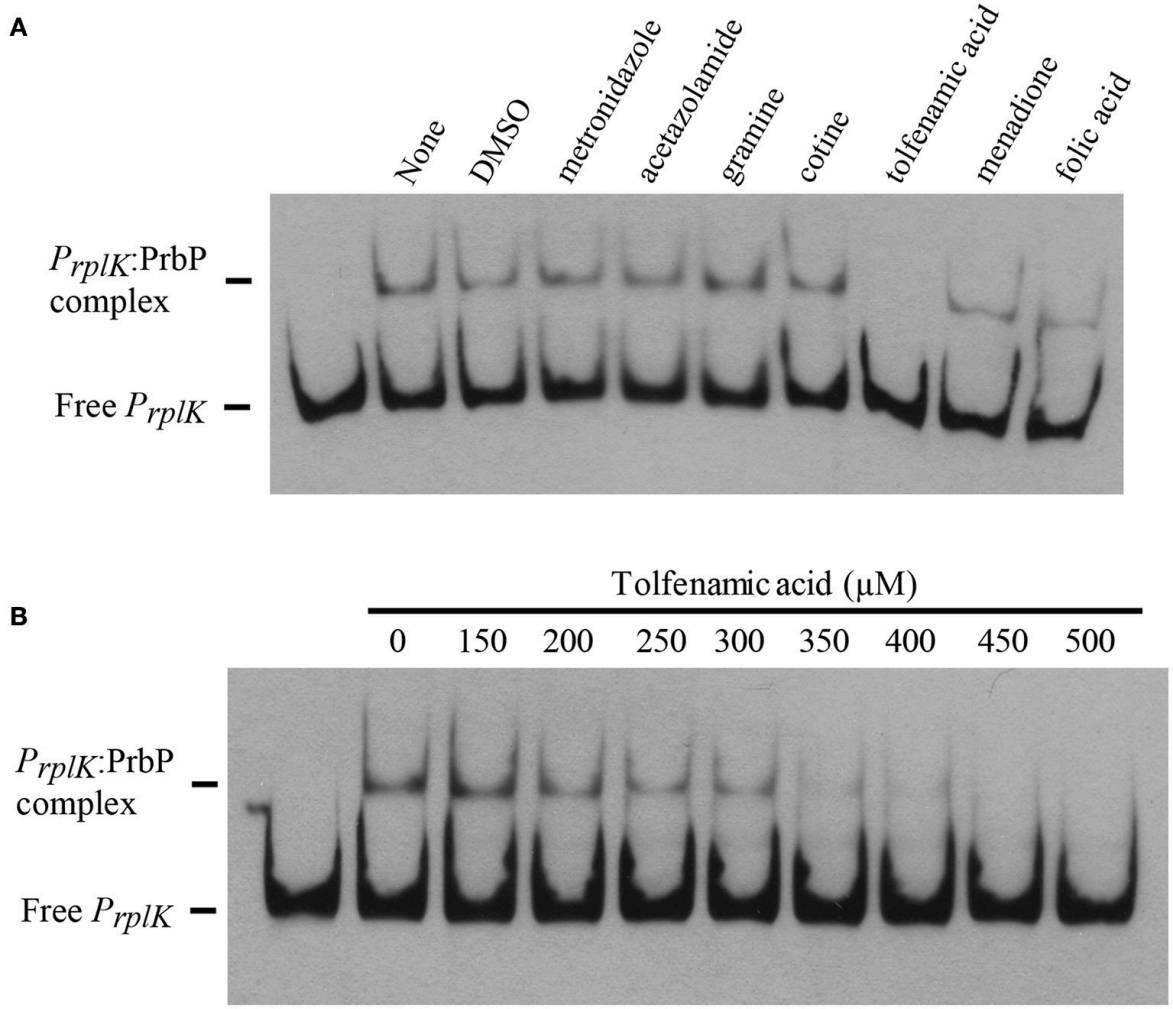
**Small molecules decrease PrbP binding to ***P***_***rplK***_**. **(A)** EMSAs were conducted with the biotin labeled probe *P*_*rplk*_ (1 ng) and PrbP (2.5 μM), in the absence (lane 2; None) and presence of 2 mM metronidazole, acetazolamide, gramine, cotine, tolfenamic acid, menadione, or folic acid. DMSO (5% final concentration) was ran as a solvent control (lane 3). No protein was added to the first lane. **(B)** EMSAs were conducted with probe *P*_*rplK*_ (1 ng) and increasing concentrations (0–500 μM) of tolfenamic acid, as indicated on top of the panel. PrbP was maintained at 2.5 μM. No protein was added to the first lane.

EMSA were performed with PrbP in the presence and absence of the small molecules identified by differential scanning fluorimetry. The promoter region of *rplK* (*P*_*rplK*_) was used as the target DNA in all assays. Of the compounds tested, tolfenamic acid was the only effective inhibitor of interactions between PrbP and *P*_*rplK*_ (Figure 5A). Complete disruption of the PrbP:*P*_*rplK*_ complex was observed with 350 μM tolfenamic acid (Figure 5B). Metronidazole, ronidazole, acetazolamide, gramine, cotine, menadione, and folic acid had no effect on the PrbP:*P*_*rplK*_ complex when present at concentrations up to 2 mM.

### Small molecules as therapeutics against *L. asiaticus*

**Figure 6:**
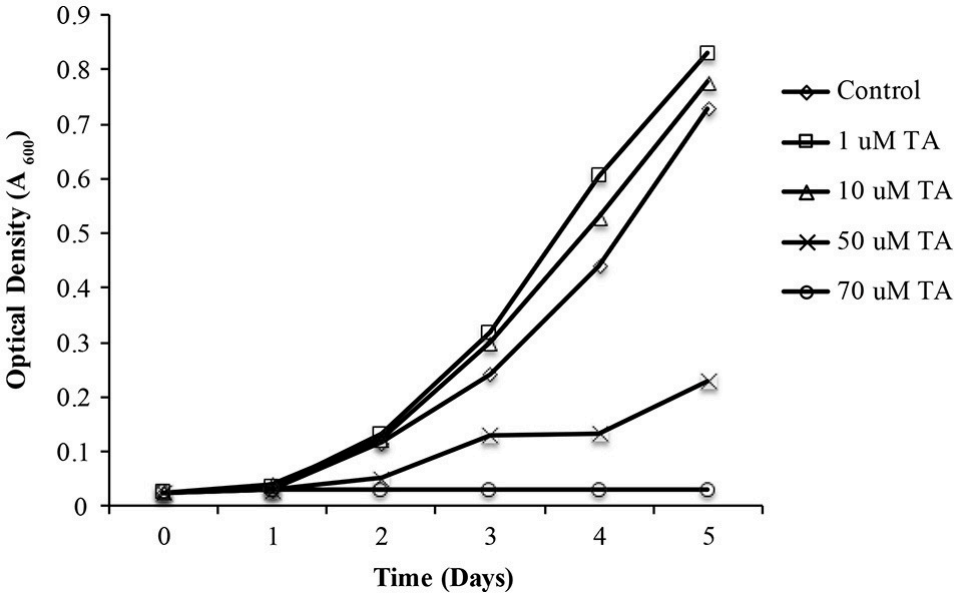
**The effect of tolfenamic acid on ***Liberibacter crescens*** growth**. *L. crescens* BT-1 was cultured at 25°C with moderate aeration (150 RPM), in modified BM7 media, with increasing concentrations (0–70 μM) of tolfenamic acid as indicated: **(**♢**)** Control without TA; **(**□**)** 1 μM TA; **(**Δ**)** 10 μM TA; **(X)** 50 μM TA; **(O)** 70 μM TA. Bacterial growth was determined by optical density at 600 nm (A_600_), over a period of 5 days.

As propagation of *L. asiaticus* still remains elusive under laboratory conditions, we used the culturable close relative *L. crescens* to determine the antimicrobial activity of metronidazole, dimetridazole, ronidazole, and tolfenamic acid. It was found that metronidazole, dimetridazole and ronidazole were not inhibitory at concentrations up to 500 μM. When combined with the results of the bacterial two hybrid system, these results suggest that interactions between PrbP and RNAP may not be critical for the persistence of *L. crescens* under the conditions tested. Conversely, growth inhibition of *L. crescens* was observed in presence of tolfenamic acid (70 μM), indicating interactions between PrbP and DNA are indeed essential for the survival of *L. crescens* (Figure 6).

**Figure 7:**
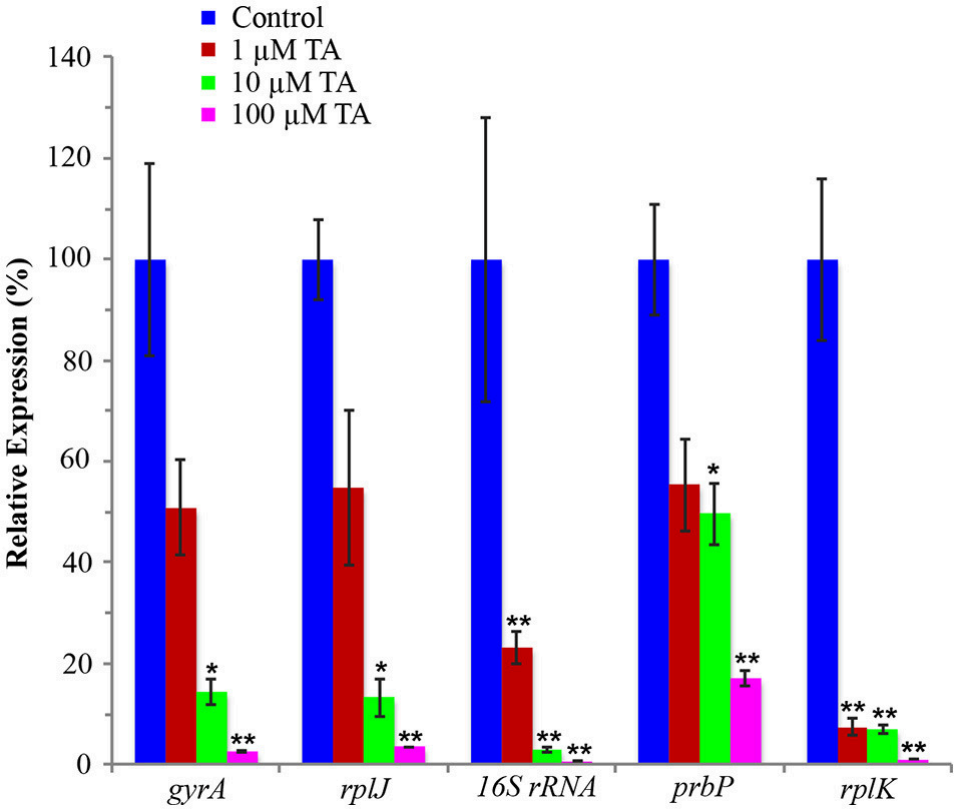
**Tolfenamic acid modulates the activity of PrbP ***in vivo*****. The effect of tolfenamic acid on the transcriptional activity of *L. asiaticus*. The expression levels of *gyrA, rplJ*, 16S RNA, *prbP* and *rplK* were assessed after 24 h. Tolfenamic acid (TA) was tested at increasing concentrations (0–100 μM) as indicated: (red) 1 μM TA, (green) 10 μM TA, (magenta) 100 μM TA. Control samples (blue) were treated with buffer only. (^*^p < 0.05; ^**^p < 0.005). The plant gene *cox2* was used to normalize the expression values between samples.

An infected leaf assay was used to assess the efficacy of tolfenamic acid against *L. asiaticus, in vitro* (Pagliai et al., 2014). Leaves were collected from Valencia Orange (*C. sinensis*) trees infected with *L. asiaticus*. Upon collection, leaves were immediately immersed in 1, 10, or 100 μM tolfenamic acid. Following incubation, each sample was analyzed by qRT-PCR to determine the relative change in gene expression for 16S rRNA, DNA gyrase subunit A, L10 ribosomal, PrbP and RplK protein (encoded by *CLIBASIA_r05785, gyrA, rplJ, prbP*, and *rplK*, respectively) as viability parameters for *L. asiaticus* (Pagliai et al., 2014). Due to the variability in bacterial load, samples were normalized to the plant gene *cox2* and *18S rRNA*. Samples incubated with 10 or 100 μM tolfenamic acid showed a significant (*p* < 0.05) decrease in the expression of all five genes (Figure 7). These results suggest that tolfenamic acid may be inhibitory to *L. asiaticus* at 10 and 100 μM.

The addition of 1 μM tolfenamic acid did not have a significant effect on the expression levels of *gyrA, prbP*, or *rplJ*, however, a significant (*p* < 0.005) decrease in expression was observed for *rplK* and 16S rRNA (93 and 77% reduction, respectively). These results indicate that PrbP is a transcriptional activator for *rplK* and the 16S ribosomal genes in *L. asiaticus*. Additionally, these results indicate that expression of *prbP* is not auto-regulated at the level of transcription, in a manner dependent on DNA binding.

### Use of tolfenamic acid as antimicrobial in HLB-infected citrus seedlings

**Figure 8:**
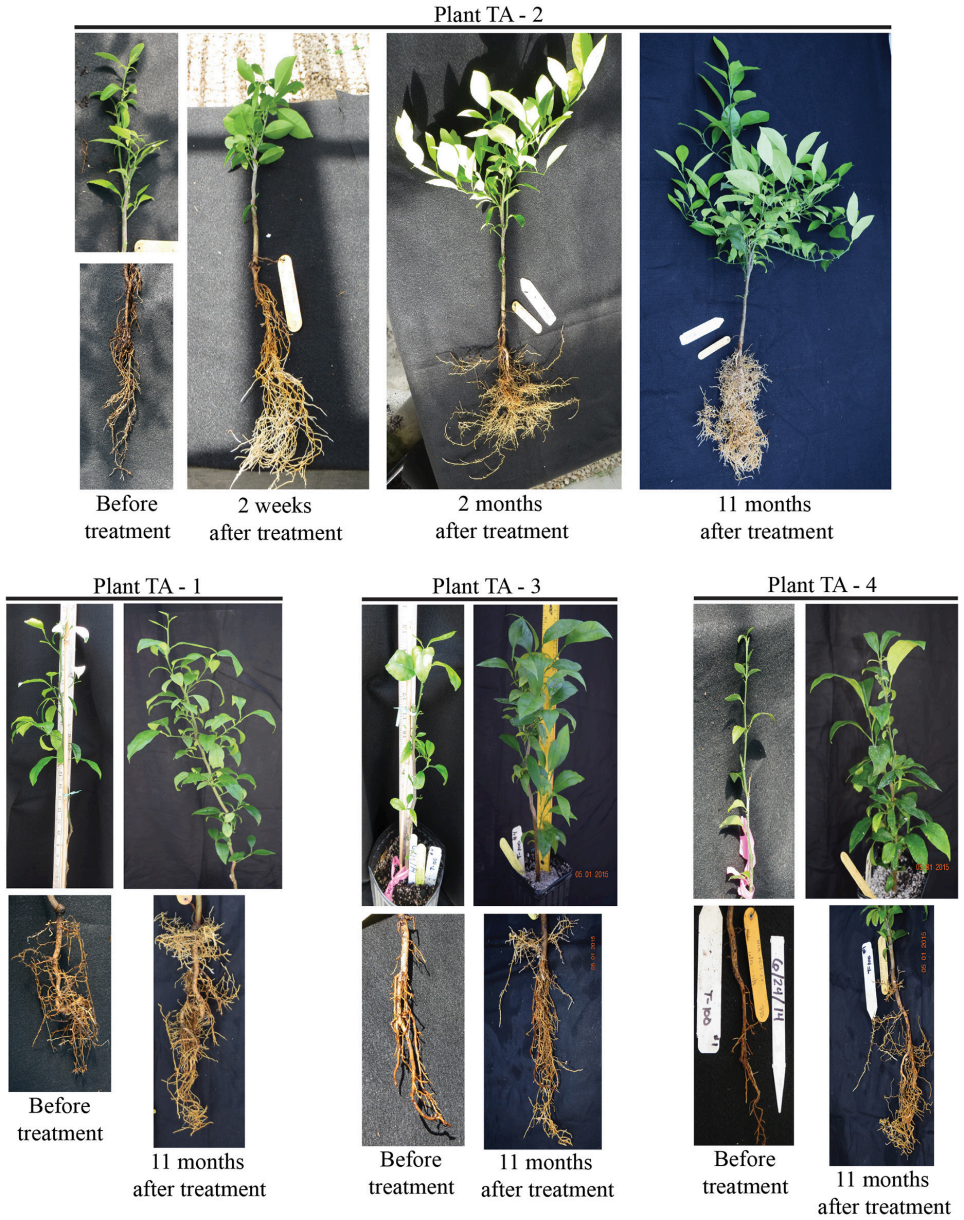
**Tolfenamic acid is an effective antimicrobial compound against ***L. asiaticus*** in HLB-infected citrus seedlings**. Recovery of root and canopy tissue in *L. asiaticus*-infected seedling TA-2 (upper panel) after treatment with 100 μM tolfenamic acid. The canopy and root tissue was photographed before treatment with tolfenamic acid, 2 weeks after treatment, 2 months after treatment, and 11 months after treatment. *L. asiaticus*-infected seedlings TA-1, TA-3, and TA-4 are shown in the lower panel. Photographs were taken before treatment began and 11 months after treatment.

**Figure 9:**
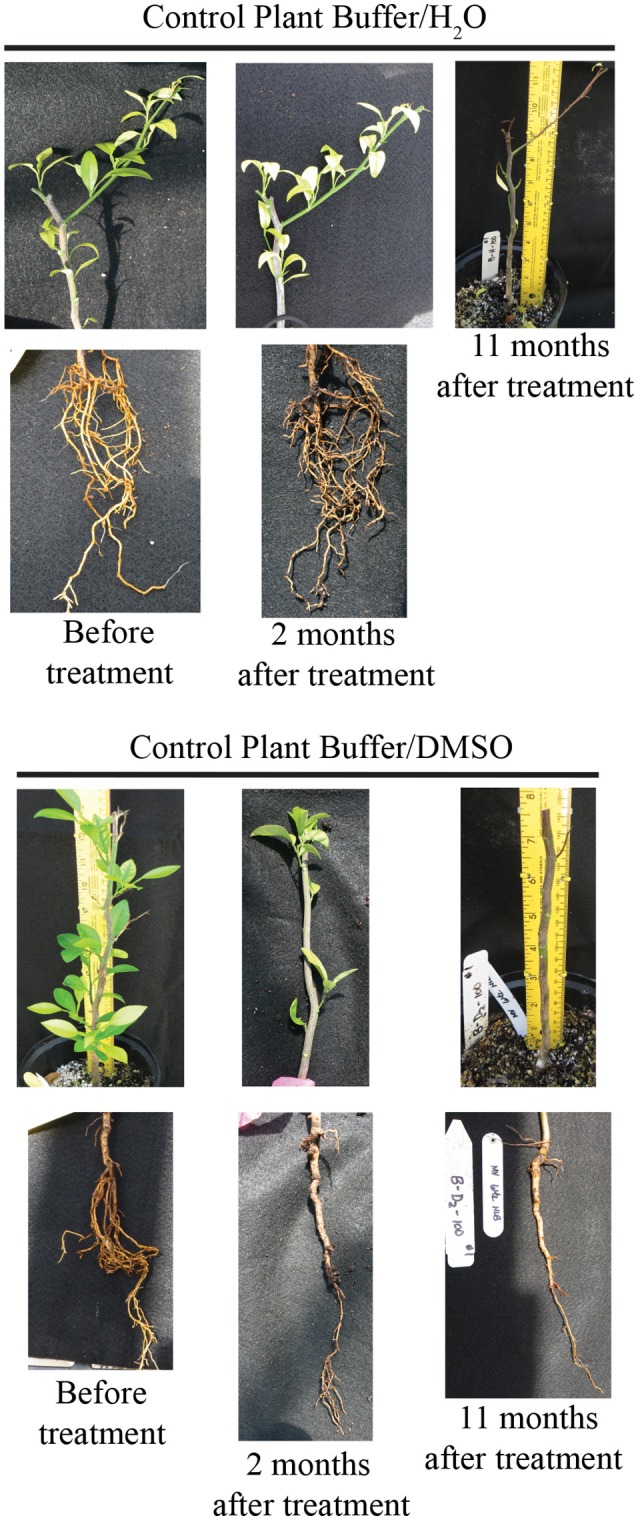
**Photographs of control seedlings (***n*** = 4) treated with buffer vehicle (10 mM Tris, 0.1% DMSO)**. Seedlings were photographed before treatment began, and post treatment (for up to 11 months).

Prior to testing the efficacy of tolfenamic acid in *L. asiaticus* infected citrus trees, a phytotoxicity assessment was performed using healthy 12 month-old citrus seedlings. Following 6 months of treatment with tolfenamic acid (1, 10, and 100 μM), no phytotoxic effects were observed in any of the treatment groups. The efficacy of tolfenamic acid was subsequently determined in *L. asiaticus*-infected citrus seedlings (*Citrus sinensis*, “Valencia”). Seedlings were infected with *L. asiaticus* (via grafting) and maintained in a greenhouse for 12 months to allow the infection to spread throughout the entire plant. Each plant had symptoms of advanced *L. asiaticus* infection and the presence of *L. asiaticus* was confirmed by PCR prior to beginning treatments with tolfenamic acid (Figures 8, 9).

**Figure 10:**
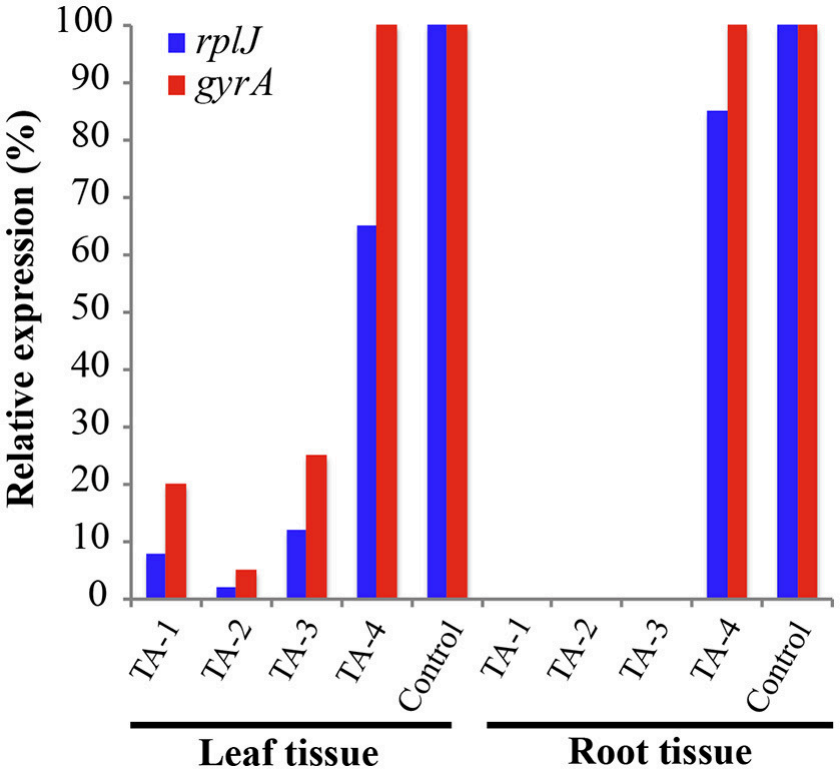
**Gene expression in leaf and root tissue 11 months after treatment with tolfenamic acid**. *L. asiaticus* mRNA levels for *rplJ* (blue) and *gyrA* (red) were determined in fresh tissue collected from treated seedlings (TA-1, TA-2, TA-3, and TA-4) and compared to the untreated controls. The plant gene *cox2* was used to normalize the expression values between samples. The lower mRNA expression levels observed in treated plants are indicative of reduced *L. asiaticus* infection. No amplification was observed in root samples from plants TA-1, TA-2, and TA-3.

Tolfenamic acid (100 μM) was administered to infected seedlings via root soaking and as a foliar spray (to saturation). Two applications were made over a period of 2 weeks. Four infected seedlings were also maintained under the same conditions as a control. Each plant was examined and photographed every 2 weeks to monitor the canopy and root tissue for signs of recovery or disease progression. New root tissue was observed in several of the treated plants 2 weeks after treatment. Two months after treatment, healthy new flush growth began to push on several of the treated plants, and continued to grow over the next 11 months with no visible signs of infection (Figure 8). Eleven months after treatments were applied, three of the four plants treated with tolfenamic acid showed clear signs of recovery in both root and canopy tissues (Figure 8). In contrast, in the control group treated with the buffer vehicle only, three out of the four plants died (Figure 9). In the treated plants, qRT-PCR analysis of root tissue revealed the absence of *L. asiaticus* infection in plants TA-1, TA-2, and TA-3 while plant TA-4 showed only a 10% reduction in the expression of *rplJ* (Figure 10). A significant drop (80–95% reduction) in *L. asiaticus* titer was also observed in the canopy tissue of 75% of the treated plants (Figure 10).

## Discussion

Due to the extended timeframe and financial obligations associated with the development and approval of new therapeutic compounds, drug repurposing has become an appealing approach to drug discovery; the availability of pharmacokinetic and pharmacodynamic data can significantly reduce research costs, and expedite the approval process. For these reasons, we elected to use a library of FDA approved compounds as a starting point for screening protein targets. The absence of putative pathogenicity determinants in *L. asiaticus*, prompted the investigation of unconventional proteins as targets for the development of therapeutic strategies against this devastating pathogen. The *in silico* analyses revealed a small number of simple regulatory mechanisms in the *L. asiaticus* genome, which enable its survival during the changing growth conditions encountered in two remarkably different hosts, the psyllid and the citrus plant. As a consequence, the transcriptional regulation of several metabolic pathways must be achieved with only a few transcription factors. Based on these observations, the inactivation of a transcription factor may result in pleiotropic effects, influencing the ability for *L. asiaticus* to survive within the citrus host.

CarD, a low identity homolog to PrbP, is an essential RNA polymerase binding protein in *Mycobacterium* species. It is involved in pathogenesis, persistence, cell viability, and resistance to both antibiotics and stress (Stallings et al., 2009; Srivastava et al., 2013). CarD interacts with both the β-subunit of the RNA polymerase and, non-specifically, with DNA sequences in the promoter region of several genes (Srivastava et al., 2013). In *L. asiaticus*, PrbP also showed interaction with RNAP and DNA. We also found that PrbP was capable of differentially binding the *rplK* promoter. The putative binding site in the *rplK* promoter is located 31 bp upstream from the translation start site. This binding location is in agreement with the role of PrbP as a transcriptional activator that may function to stabilize the open promotor complex.

The transcription of rRNA is one of the rate-limiting steps controlling cell growth under ideal and stress conditions; during this process, formation of stable RNAP/open-promotor complexes is the most time consuming step (Zhou and Jin, 1998; Bartlett et al., 2000; Jin et al., 2012; Häkkinen et al., 2013). In *Mycobacterium*, CarD was recently found to increase the formation of RNAP/open promoter complexes, and stabilize RNAP open complexes by preventing transcription bubble collapse (Davis et al., 2015; Rammohan et al., 2015). Point mutations in CarD that weaken interactions with RNAP are detrimental in *M. tuberculosis*, resulting in reduced viability and increased sensitivity to antibiotics and oxidative stress (Weiss et al., 2012). As such, we hypothesized that the inactivation/inhibition of PrbP would cause a decrease in transcript production in *L. asiaticus*, potentially resulting in decreased viability and persistence within the host.

While several compounds were found to modulate interactions between PrbP and RNA polymerase, tolfenamic acid was the only small molecule that decreased interactions between PrbP and DNA (Figure 5). The effect of these chemicals was also tested *in vivo*, where tolfenamic acid was found to be the only small molecule that affected the overall transcriptional activity of *L. crescens*. The results suggest that the ligand specificity for disruption of the PrbP:*P*_*rplK*_ complex is more stringent than the binding specificity for ligands that disrupt interactions between PrbP and RNAP.

To confirm the effect of tolfenamic acid *in planta*, tolfenamic acid was applied by root soaking and foliar spray to *L. asiaticus* infected citrus seedlings in a greenhouse setting. Each seedling was confirmed to harbor viable, *L. asiaticus* cells for at least 6 months prior to beginning treatment. In addition to testing positive for *L. asiaticus*, each plant also displayed severe signs of infection, including blotchy mottle, yellowing shoots, and severe damage to the root system. After the initial treatment, the first sign of recovery in treated seedlings was the development of new root growth. Rapid recovery of the root system was observed in 75% of seedlings that were treated with tolfenamic acid (Figures 8, 10). Although several plants showed improved root growth in as little as 14 days after treatment, the canopy tissue was slower to recover (5–11 months before substantial amounts of new growth was observed).

The extent of vascular damage caused by the presence of *L. asiaticus* may affect the rate that compounds (chemical treatments) are distributed throughout the canopy, and thus the rate at which each plant recovers. Previous studies have shown that HLB significantly affects the vascular flow and exchange of nutrients between root and canopy tissue (Etxeberria et al., 2009; Koh et al., 2012). Factors contributing to the reduction in vascular flow include the accumulation of starch within the xylem and phloem, the blockage of sieve elements by callose formation and bacterial cell debris, and compartmentalization as the tree attempts to isolate the pathogen (Kim et al., 2009; Koh et al., 2012; Aritua et al., 2013). During our analysis of tissue samples collected from HLB-infected seedlings, the highest expression levels of *L. asiaticus* genes (post-treatment) was observed in tissue collected from underdeveloped regions of the canopy, where leaf growth was stagnate. Since the static growth of these branches is indicative of severely obstructed vascular flow, it is possible that the transport of small molecules (from the injection site) to these regions of the tree was insufficient to deliver enough chemical to completely inhibit the growth of *L. asiaticus*. As such, the treatment of highly infected citrus trees may require repeat injections or a combined application method that will facilitate even distribution throughout the canopy tissue (such as a foliar spray with adjuvants). Combined treatments with small molecules and thermotherapy may also be a viable option, as more efficient methods of heat treatment are developed.

There are very few antibiotics approved for use in the treatment of plant diseases, and of those, only streptomycin and tetracycline have shown minimal success in the treatment of HLB, with tetracycline having phytotoxic effects at the concentrations required for treatment of *L. asiaticus*. The combined use of penicillin and streptomycin showed a suppressive effect on *L. asiaticus* (Zhang et al., 2011), however, the use of β-lactam antibiotics is highly regulated and not approved for use in agriculture. As such, the use of small molecules is a promising alternative to combat *L. asiaticus*.

In this study, we were able to confirm that tolfenamic acid is an effective inhibitor of *L. asiaticus in planta*. While additional studies are needed to fully understand the recovery process of treated trees, these encouraging results indicate the potential for tolfenamic acid to be used as a systemic antimicrobial therapy for HLB.

## Author contributions

CLG, FP, LP, LB, and MT conducted the experiments and analyzed the results. CLG, GL, and CFG conceived the idea for the project, analyzed the results, and wrote the paper.

### Conflict of interest statement

The authors declare that the research was conducted in the absence of any commercial or financial relationships that could be construed as a potential conflict of interest. A patent application has been submitted for the use of tolfenamic acid for the treatment of HLB.

## Abbreviations

EMSA: electrophoretic mobility shift assay.
